# Tanshinone Capsules Combined With Prednisone for Facial Seborrheic Dermatitis: A Systematic Review and Meta-Analysis of Randomized Clinical Trials

**DOI:** 10.3389/fmed.2022.816419

**Published:** 2022-04-29

**Authors:** Qiang Fu, Mengya Huang, Lei Tang, Qi Zheng, Fujun Huang, Xun Zhou, Shumei Wang

**Affiliations:** ^1^College of Traditional Chinese Medicine, Chongqing Medical University, Chongqing, China; ^2^Department of Dermatology and Cosmetology, Chongqing Traditional Chinese Medicine Hospital, Chongqing, China

**Keywords:** facial seborrheic dermatitis, tanshinone, triamcinolone acetonide and econazole cream, prednisone, meta-analysis

## Abstract

**Background:**

Facial seborrheic dermatitis (FSD), also called facial seborrheic eczema, is a common disease affecting both male and female patients worldwide. Tanshinone is the main bioactive component extracted from the Traditional Chinese Medicine *Salvia miltiorrhiza Bunge*, which is widely used in treating skin inflammatory diseases. It is necessary to evaluate the clinical evidence for tanshinone capsule treatment of FSD. This study aimed to evaluate the safety and effectiveness of tanshinone capsules combined with prednisone in the treatment of facial seborrheic dermatitis and to provide evidence for clinical practice.

**Methods:**

Studies were searched in PubMed, the Cochrane Library, the Chinese Biomedical Literature Database, the China National Knowledge Infrastructure, the Chinese Scientific Journal Database, and WanFang Database before October 2021. We also searched for randomized controlled clinical trials (RCT) of tanshinone capsules combined with prednisone on facial seborrheic dermatitis. The meta-analysis was conducted according to the guidelines of the Cochrane Handbook. Two reviewers regulated the research selection, data extraction, and risk of bias assessment, respectively, and a third reviewer was used for consulting when necessary. Review Manager Software 5.3 was used for meta-analysis.

**Results:**

A total of 10 RCTs with 916 participants were included. Nine studies reported total effectiveness, five studies reported symptom score, seven studies reported adverse events, and four studies reported recurrence rate. The duration of treatment was 4 to 8 weeks. Combination therapy showed better clinical effects compared to the prednisone (OR: 5.82; 95% CI: 3.53, 9.59; *p* < 0.00001). Combination therapy could repair skin lesions (MD: −0.40; 95% CI: −0.51, −0.30; *p* < 0.00001), reduce skin erythema (MD: −0.58, 95% CI: −0.67, −0.49; *p* < 0.00001), relieve skin itch (MD: −0.70; 95% CI −0.77, −0.63; *p* < 0.00001), and desquamation score (MD: −0.64; 95% CI: −0.71, −0.56; *p* < 0.00001). Furthermore, combination therapy could reduce adverse events (OR: 0.46; 95% CI: 0.26, 0.84; *p* = 0.01) and control recurrence rate (OR: 0.22; 95% CI: 0.13, 0.36; *p* < 0.00001).

**Conclusions:**

Compared with prednisone, tanshinone capsules combined with prednisone may be effective in the treatment of facial seborrheic dermatitis. However, due to the high risk and ambiguity of bias in the included trials, the conclusion of this study must be interpreted carefully.

## Introduction

Facial seborrheic dermatitis (FSD), also called facial seborrheic eczema, belongs to the category of skin appendage diseases. It is a chronic superficial papulosquamous skin disease characterized by hypersecretion of sebaceous glands and increased oil secretion, with scaling or pruritus (1). Seborrheic dermatitis affects 3–10% of the general population (2), and the incidence rate of this disease is as high as 30–83% in HIV patients (3). The lesions are typically located on the face (87.7%) due to the face having the most sebaceous glands involving the forehead, eyebrows, glabella, and nasolabial folds (4). The clinical symptoms of seborrheic dermatitis are erythema, pruritus, and other inflammatory manifestations, or non-inflammatory manifestations like furfuraceous desquamation. Although FSD is not fatal, facial skin lesions seriously affect a person's social image and reduce their quality of life. It not only brings a high economic burden to Asian countries but also puts severe pressure on people's mental health (5, 6). In China, about 48.1% of patients have serious emotional problems (7).

At present, there is still a lack of understanding of the pathogenesis of FSD. Although traditional drug treatment has some effects, unfortunately, no widespread completely recognized treatment exists for all situations. FSD is a chronic progressive disease, long-term use of drugs will inevitably bring some side effects and drug resistance. Tanshinone, a representative natural product, is the main compound extracted from the dried root and rhizome of *Salvia miltiorrhiza Bunge [Lamiaceae]*, which has the effect of promoting blood circulation, removing blood stasis, detumescence and relieving pain, regulating menstruation, calming nerves, and maintaining endocrine balance (8). The main active ingredient of tanshinone capsules is cryptotanshinone (9), with the in-depth study of more and more identified analogs, tanshinone has shown a variety of biological activities, including anti-apoptosis, anti-inflammatory, antioxidant stress pathways, and phytoestrogenic activity (10). Tanshinone is often used in the treatment of skin inflammatory diseases and there are plenty of reports on the clinical research of FSD. However, there is no systematic evaluation of tanshinone capsules combined with prednisone in the treatment of FSD, which makes it impossible for us to understand the effect of combination therapy. Therefore, this study collates clinical research studies and intends to systematically evaluate the efficacy and safety of tanshinone capsules combined with prednisone in the treatment of FSD, and provides a reference for clinical practice.

## Materials and Methods

### Inclusion Criteria

Types of studies: only RCTs were included, other studies such as case reports and animal experiments were excluded. Types of participants: the study considered participants aged 18 years or older given the diagnosis of FSD defined by clear diagnostic criteria, irrespective of their gender, severity, education, and disease duration. Type of interventions: the intervention was a tanshinone capsule combined with prednisone, and the original literature required a clear description of the manufacturer, dose, and usage of tanshinone and prednisone. Types of controls: the control measure was triamcinolone acetonide and econazole cream (trade name “prednisone”), with clear reporting of the method of medication, dosage, and course of treatment. Types of outcome measures: the primary outcome measures included the effective rate, secondary outcome measures included symptom score (symptoms included erythema, itchy, furfuraceous desquamation, and lesions), recurrence rate, and incidence of any adverse events (AEs).

### Exclusion Criteria

The exclusion criteria contain the following items: first, the literature related to the same study, and duplicate publications. Second, unable to get literature for available data or full text through various means. Last, the outcome indexes were not counted according to the same evaluation criteria.

### Search Strategy

The following electronic databases were searched: PubMed, the Cochrane Library, the Chinese Biomedical Literature Database (CBM), China National Knowledge Infrastructure (CNKI), Technology Journal Database, and China Science and the Wan Fang Databases (WF). The last search for all databases was updated to October 31, 2021, and studies published in Chinese or English publications were included. The search terms include “facial seborrheic dermatitis,” “seborrheic dermatitis,” “tanshinone,” “triamcinolone acetonide and econazole cream,” and “prednisone,” the search strategies are adjusted according to the characteristics of different databases.

### Study Selection and Data Management

Initially, two researchers screened the literature independently and imported the retrieved records into Endnote X7 to sort and remove duplicates. They then read the abstract and the title and eliminated literature that did not conform to the theme. They also reviewed the full text, further screened it according to the inclusion and exclusion criteria, and recorded the reasons for the deletion of literature. Ultimately, according to the updated guidelines for reporting parallel group randomized trials (11), the data extraction including the first-author, publication year, sample size, age of patients, gender, intervention measures, treatment methods, and treatment time. The extracted data was cross-checked, and a third reviewer was consulted when necessary.

### Risk of Bias Assessment

The quality of the studies was assessed according to the “Risk of bias” tool from the Cochrane Handbook (12). The evaluation includes seven items: random sequence generation, allocation concealment, blinding method, incomplete data assessment, selective reporting, and other biases. Evidence quality was divided into three categories: “low bias risk,” “unclear bias risk,” and “high bias risk.” Then the evaluation results were shown by the bias risk assessment chart drawn by Review Manager 5.3.

### Data Synthesis and Analysis

Where there were multiple eligible intervention groups, data were combined from active treatment arms into one group to create a single pair-wise comparison as per the Cochrane guidelines (13). RevMan 5.3 software was used to analyze the data. Binary variables were statistically analyzed by odds ratio (OR) and continuous variables were statistically analyzed by mean difference (MD). Ninety-five percentage confidence interval (CI) was used to evaluate each effect index. When the 95% CI of MD does not include 0 or the 95% CI of or does not include 1, the difference between the two intervention methods was statistically significant (*p* < 0.05). The heterogeneity of the study was evaluated according to the guidelines of revman5.3 software, and the value of *I*^2^ was used for heterogeneity calculations. The random-effects model was used for analysis if *I*^2^ ≥ 50%, otherwise, the fixed effects model was used for analysis when *I*^2^ < 50%, and the evaluation results are shown in forest maps. When the results show substantial heterogeneity or considerable heterogeneity, subgroup analysis and sensitivity analysis were made to look for the possible causes. Finally, a bias test was performed on the effective rate, and the results are displayed in a funnel chart.

## Results

### Search Result

**Figure 1:**
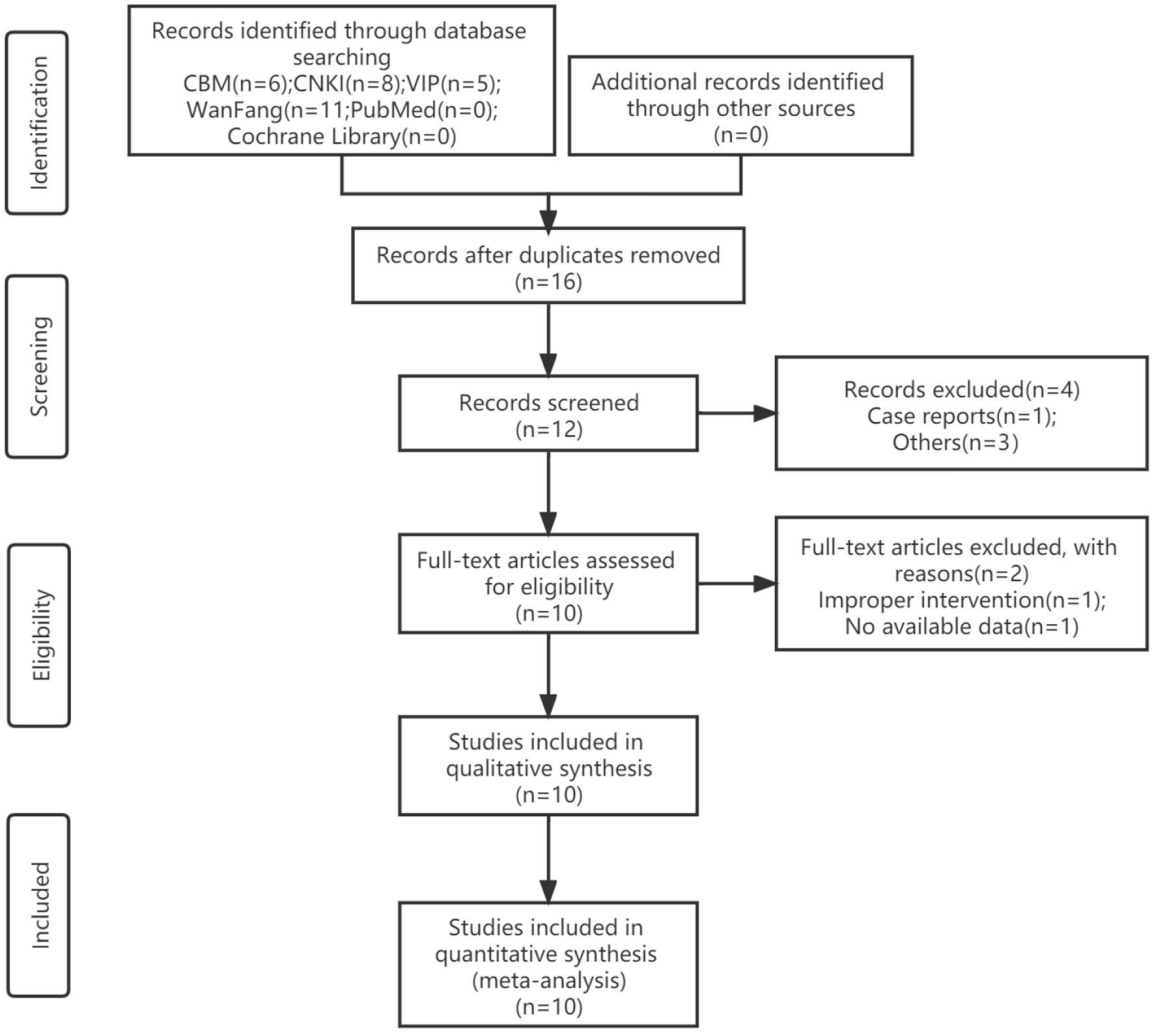
Flow diagram of study selection and identification.

A total of 30 studies were retrieved, and 18 remained after screening titles and abstracts. We read the full text of these 12 studies, and by excluding 2 studies finally included 10 articles (14–23). All trials were designed as two eligible arms and used the parallel-group design. The screening process is shown in Figure 1.

### The Characteristics of Included Trials

The trials included 10 RCTs with 916 participants, 460 in the experimental group, and 456 in the control group. All of the trials were conducted in China and published in Chinese. All of the trials were compared to tanshinone combined with prednisone vs. using the prednisone alone. Nine trials reported the treatment duration was 4 weeks, and one study was 8 weeks (16). The participants were aged 18–56 years. The course of illness was 2 months to 6 years. The dosage of tanshinone in all test groups was 1 g/time, 3 times/day, and the dosage of pevisone was 2 times/day. Nine trials reported the clinical efficacy rates (14–19, 21–23), 5 trials reported symptom scores (15, 16, 19, 20, 22), 7 trials reported adverse events (14, 16, 18–21, 23), and 4 trials reported recurrence rate (14, 15, 18, 19). The characteristics of the included trials are shown in Table 1, and the quality control of tanshinone capsules is shown in Table 2.

**Table 1:** Characteristics of the included trials.

**Study ID**	**Country**	**Sample size (M/F)**	**Age (Mean ±SD), years**	**Course of disease**	**Course of treatment**	**Intervention vs. Control**	**Drug dosege**	**Outcomes**
Guan (14)	China	T:55 (21/34)	T:29.36 ± 2.59	T:2.25 ± 0.27 y	4 w	Tanshinone + Pevisone vs. Pevisone	Tanshinone: 1 g/time, 3 times/day; Pevisone: 2 times/day	Clinical effect, Adverse events, Recurrence rate
		C:55 (20/35)	C:22.12 ± 3.16	C:2.15 ± 0.26 y				
Wen et al. (15)	China	T:32 (18/14)	T:42.31 ± 3.87	T:32.51 m	4 w	Tanshinone + Pevisone vs. Pevisone	Tanshinone: 1 g/time, 3 times/day; Pevisone: 2 times/day	Clinical effect, Clinical symptom score, Recurrence rate
		C:34 (26/18)	C:41.35 ± 3.76	C:33.34 m				
Li (16)	Chin	T:55 (30/25)	T:35.68 ± 5.47	T:4.69 ± 1.28 y	8 w	Tanshinone + Pevisone vs. Pevisone	Tanshinone: 1 g/time, 3 times/day; Pevisone: 2 times/day	Clinical effect, Clinical symptom score, Adverse events
		C:55 (32/23)	C:34.14 ± 6.45	C:4.10 ± 1.14 y				
Wang (17)	China	T:41 (30/11)	T:32.50 ± 5.40	T:1.60 ± 0.40 y	4 w	Tanshinone + Pevisone vs. Pevisone	Tanshinone: 1 g/time, 3 times/day; Pevisone: 2 times/day	Clinical effect
		C:40 (30/10)	C:32.80 ± 5.10	C:1.50 ± 0.32 y				
Chen et al. (18)	China	T:44 (23/21)	T:25.12 ± 2.78	T:3.63 ± 1.01 y	4 w	Tanshinone + Pevisone vs. Pevisone	Tanshinone: 1 g/time, 3 times/day; Pevisone: 2 times/day	Clinical effect, Adverse events, Recurrence rate
		C:44 (24/20)	C:24.33 ± 3.29	C:3.63 ± 1.01 y				
Liu et al. (19)	China	T:60 (26/34)	T:25.35 ± 8.14	T:32.19 ± 11.58 y	4 w	Tanshinone + Pevisone vs. Pevisone	Tanshinone: 1 g/time, 3 times/day; Pevisone: 2 times/day	Clinical effect, Clinical symptom score, Adverse events, Recurrence rate
		C:60 (20/40)	C:25.21 ± 7.10	C:31.20 ± 14.35 y				
Qin (20)	China	T:27 (15/12)	T:27.39 ± 2.27	NR	4 w	Tanshinone + Pevisone vs. Pevisone	Tanshinone: 1 g/time, 3 times/day; Pevisone: 2 times/day	Clinical symptom score, Adverse events
		C:27 (14/13)	C:27.23 ± 2.30					
Wang et al. (21)	China	T:60 (24/36)	T:34.1 ± 5.9	T:3 m−4 y	4 w	Tanshinone + Pevisone vs. Pevisone	Tanshinone: 1 g/time,3 times/day; Pevisone: 2 times/day	Clinical effect, Adverse events
		C:60 (20/40)	C:34.6 ± 6.1	C:2 m−4 y				
Lu (22)	China	T:44 (23/21)	T:35.39 ± 4.59	NR	4 w	Tanshinone + Pevisone vs. Pevisone	Tanshinone: 1 g/time, 3 times/day; Pevisone: 2 times/day	Clinical effect, Clinical symptom score
		C:44 (25/19)	C:35.16 ± 4.87					
You (23)	China	T:42 (25/17)	T:38.23 ± 5.12	T:2.57 ± 0.72 y	4 w	Tanshinone + Pevisone vs. Pevisone	Tanshinone: 1 g/time, 3 times/day; Pevisone: 2 times/day	Clinical effect, Adverse events
		C:37 (22/15)	C:38.06 ± 5.48	C:2.62 ± 0.80 y				

*Y, year; m, month; NR, not reported; M, male; F, female; C, control; T, Test*.

**Table 2:** Quality control of Tanshinone capsules.

**Study**	**Formulation**	**Source**	**Compound, concentration**	**Quality control reported? (Y/N)**	**Chemical analysis reported (Y/N)**
Guan (14)	Tanshinone capsules	Hebei xinglong Xili Pharmaceutical Co., Ltd	Ethanol extract from Root of *Salvia miltiorrhiza Bunge [Labiatae]*, 0.25 g	Y—Prepared according to National Medical Products Administration (No. Z13020110)	N
Wen et al. (15)	Tanshinone capsules	Hebei xinglong Xili Pharmaceutical Co., Ltd	Ethanol extract from Root of *Salvia miltiorrhiza Bunge [Labiatae]*, 0.25 g	Y—Prepared according to National Medical Products Administration (No. Z13020110)	N
Li (16)	Tanshinone capsules	Hebei xinglong Xili Pharmaceutical Co., Ltd	Ethanol extract from Root of *Salvia miltiorrhiza Bunge [Labiatae]*, 0.25 g	Y—Prepared according to National Medical Products Administration (No. Z13020110)	N
Wang (17)	Tanshinone capsules	Hebei xinglong Xili Pharmaceutical Co., Ltd	Ethanol extract from Root of *Salvia miltiorrhiza Bunge [Labiatae]*, 0.25 g	Y—Prepared according to National Medical Products Administration (No. Z13020110)	N
Chen et al. (18)	Tanshinone capsules	Hebei xinglong Xili Pharmaceutical Co., Ltd	Ethanol extract from Root of *Salvia miltiorrhiza Bunge [Labiatae]*, 0.25 g	Y—Prepared according to National Medical Products Administration (No. Z13020110)	N
Liu et al. (19)	Tanshinone capsules	Hebei xinglong Xili Pharmaceutical Co., Ltd	Ethanol extract from Root of *Salvia miltiorrhiza Bunge [Labiatae]*, 0.25 g	Y—Prepared according to National Medical Products Administration (No. Z13020110)	N
Qin (20)	NR	NR	NR	NR	N
Wang et al. (21)	Tanshinone capsules	Hebei xinglong Xili Pharmaceutical Co., Ltd	Ethanol extract from Root of *Salvia miltiorrhiza Bunge [Labiatae]*, 0.25 g	Y—Prepared according to National Medical Products Administration (No. Z13020110)	N
Lu (22)	Tanshinone capsules	Hebei xinglong Xili Pharmaceutical Co., Ltd	Ethanol extract from Root of *Salvia miltiorrhiza Bunge [Labiatae]*, 0.25 g	Y—Prepared according to National Medical Products Administration (No. Z13020110)	N
You (23)	Tanshinone capsules	Hebei xinglong Xili Pharmaceutical Co., Ltd	Ethanol extract from Root of *Salvia miltiorrhiza Bunge [Labiatae]*, 0.25 g	Y - Prepared according to National Medical Products Administration (No. Z13020110)	N

### Risk of Bias in Included Trials

**Figure 2:**
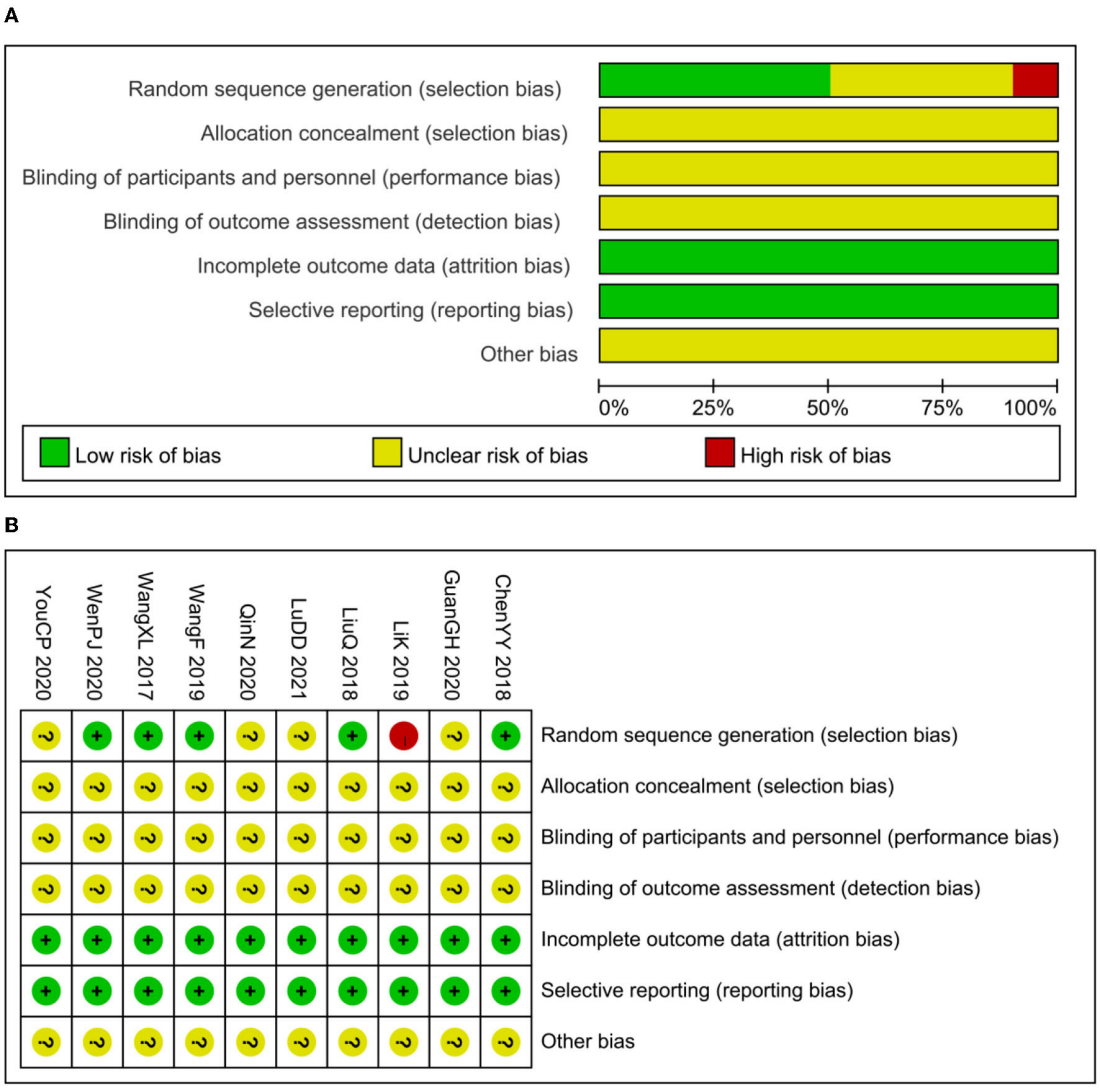
Assessment of risk of bias. **(A)** Risk of bias graph and **(B)** risk of bias summary.

Five studies reported methods of randomizing participants by using random number tables, which were considered to be a low risk of bias. In total, 4 trials mentioned randomization but did not explain the randomization method in detail (14, 20, 22, 23), which was considered to be an unclear risk of bias. Another study was considered to be high risk by grouping patients in the availability of interventions (16). None of the studies reported adequate allocation concealment not that they were blinded to participants or outcome evaluators, which was considered to be an unclear risk of bias. All studies had no patients fell off, all studies reported test indicators as planned, and there was no selective reporting of research results. It is unclear whether there were other examples of bias (Figure 2).

### Primary Outcomes

#### The Clinical Efficacy Rates

**Figure 3:**
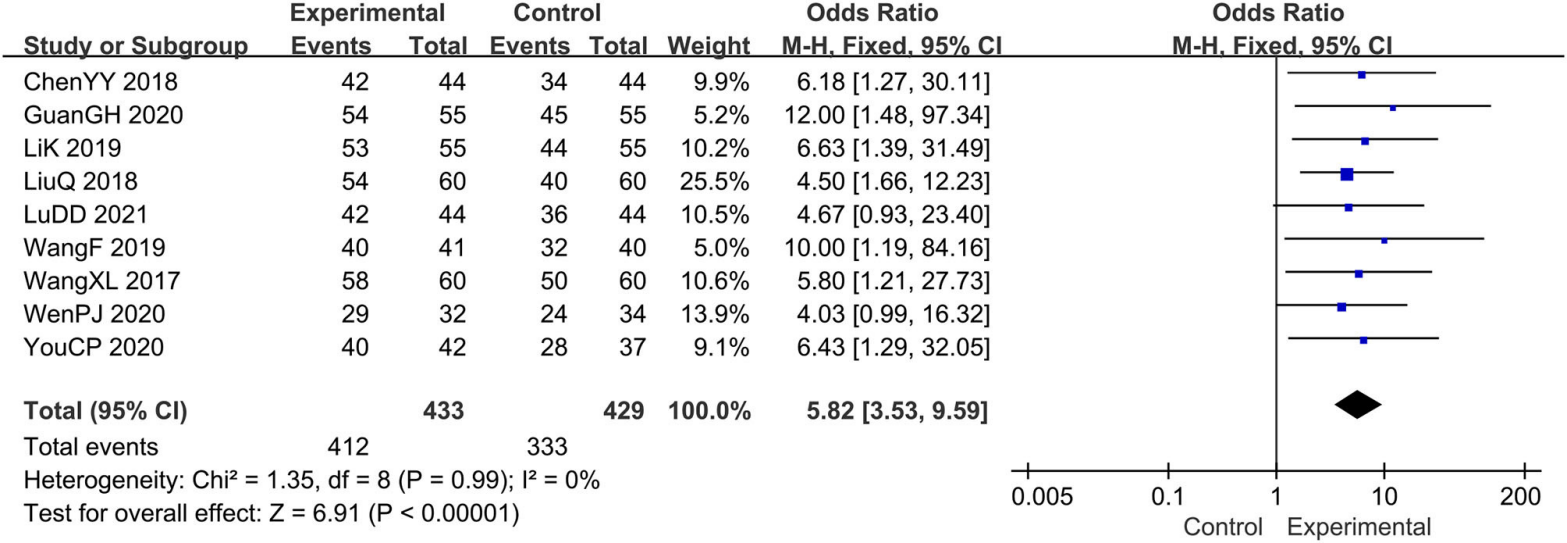
The meta-analysis for comparison of combination therapy vs. prednisone for the outcome of the clinical efficacy rates.

A total of 9 studies (14–19, 21–23) evaluated the clinical efficacy rates, comprising a total of 862 patients. Combination therapy was the experimental group, and the prednisone group was used as the control group. For the chi-square test: χ^2^ = 1.35, *I*^2^ < 1%, indicating that each study is homogeneous. Therefore, the fixed-effect model should be used for statistical analysis. The forest map generated after statistical combination formed the shape of a diamond situated to the right of the invalid line OR: 5.82; 95% CI: 3.53, 9.59; *p* < 0.00001, which means there is a statistically significant difference between the experimental group and the control group. In other words, the curative effect of combination therapy was significantly better than that with prednisone (Figure 3).

### Secondary Outcomes

#### The Score of Lesions

**Figure 4:**
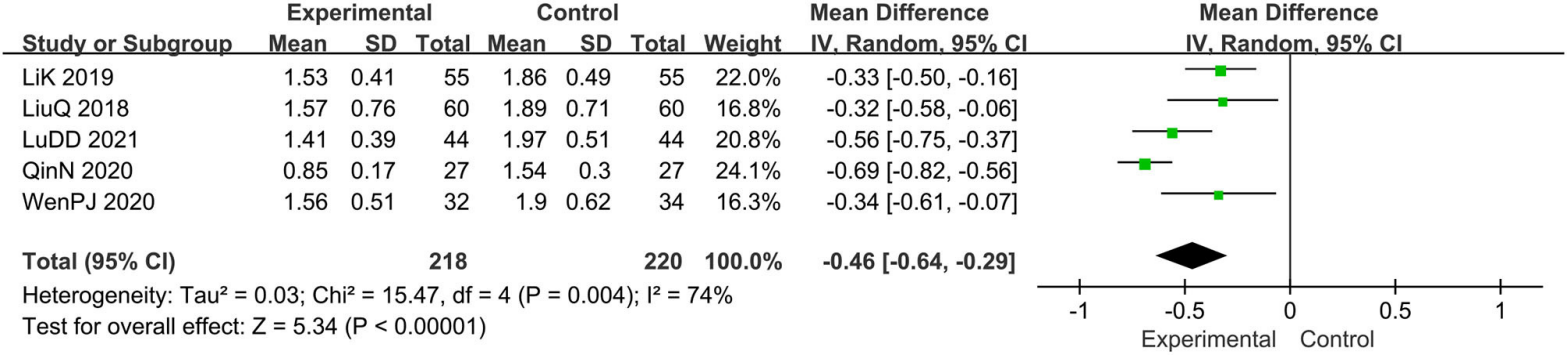
The meta-analysis for comparison of combination therapy vs. prednisone for the outcome of the score of lesions.

**Figure 5:**
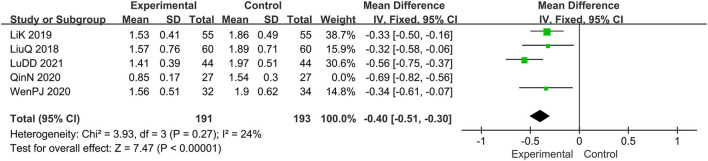
The meta-analysis for the score of lesions (without the heterogeneity study).

Five trials evaluated the score of lesions (15, 16, 19, 20, 22), comprising a total of 438 patients. The random-effect model was adopted due to the high heterogeneity (*I*^2^ = 74%, *p* = 0.004). Results show that relative to the control group, the experimental group significantly alleviated the skin lesions, MD: −0.46, 95 CI: −0.64, −0.29; *p* = 0.004(<0.05; Figure 4). To reduce heterogeneity, we eliminated the literature one by one and found that the overall heterogeneity was low after removing Qin N2020(*I*^2^ = 24%, *p* = 0.27), Therefore, the fixed-effect model should be used for subsequent statistical analysis. The results showed that MD: −0.40; 95% CI: −0.51, −0.30; *p* < 0.00001, which also means that combination therapy is more effective (Figure 5).

#### The Score of Pruritus

**Figure 6:**
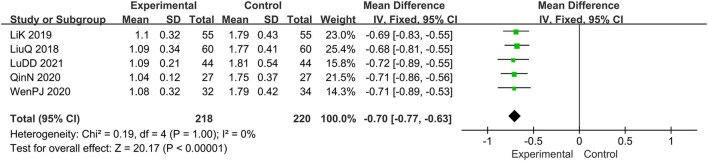
The meta-analysis for comparison of combination therapy vs. prednisone for the outcome of the score of pruritus.

Five trials evaluated the score of pruritus (15, 16, 19, 20, 22), comprising a total of 438 patients. The chi-square test showed *I*^2^ = 0%, indicating the heterogeneity was low. Therefore, the fixed effect model should be used for statistical analysis. The forest map generated after statistical combination formed the shape of a diamond situated to the left of the invalid line, MD: −0.70; 95% CI −0.77, −0.63; *p* < 0.00001, thus, the curative effect of the experimental group was significantly better than that with the control group (Figure 6).

#### The Score of Desquamation

**Figure 7:**
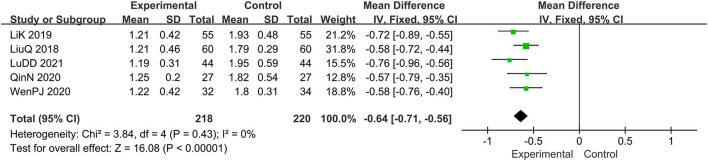
The meta-analysis for comparison of combination therapy vs. prednisone for the outcome of the score of desquamation.

Five trials evaluated the score of desquamation (15, 16, 19, 20, 22), comprising a total of 438 patients. The fixed-effect model was adopted due to the low heterogeneity (*I*^2^ = 0%, *p* = 0.43). Results showed that relative to the prednisone group, combined with tanshinone significantly reduced the score of desquamation, MD: −0.64; 95% CI: −0.71, −0.56; *p* < 0.00001 (Figure 7).

#### The Score of Erythema Symptoms

**Figure 8:**
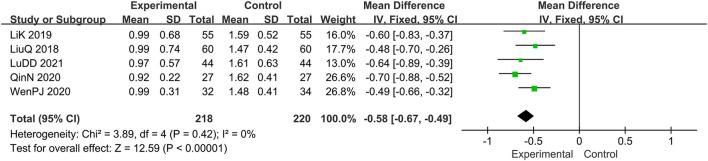
The meta-analysis for comparison of combination therapy vs. prednisone for the outcome of the score of erythema symptoms.

Five trials evaluated the erythema symptoms (15, 16, 19, 20, 22), comprising a total of 438 patients. The fixed-effect model was adopted due to the low heterogeneity (*I*^2^ = 0%, *p* = 0.42). It was found that the experimental group has a better clinical effect in alleviating erythema symptoms, MD: −0.58, 95% CI: −0.67, −0.49; *p* < 0.00001 (Figure 8).

### The Adverse Events

**Figure 9:**
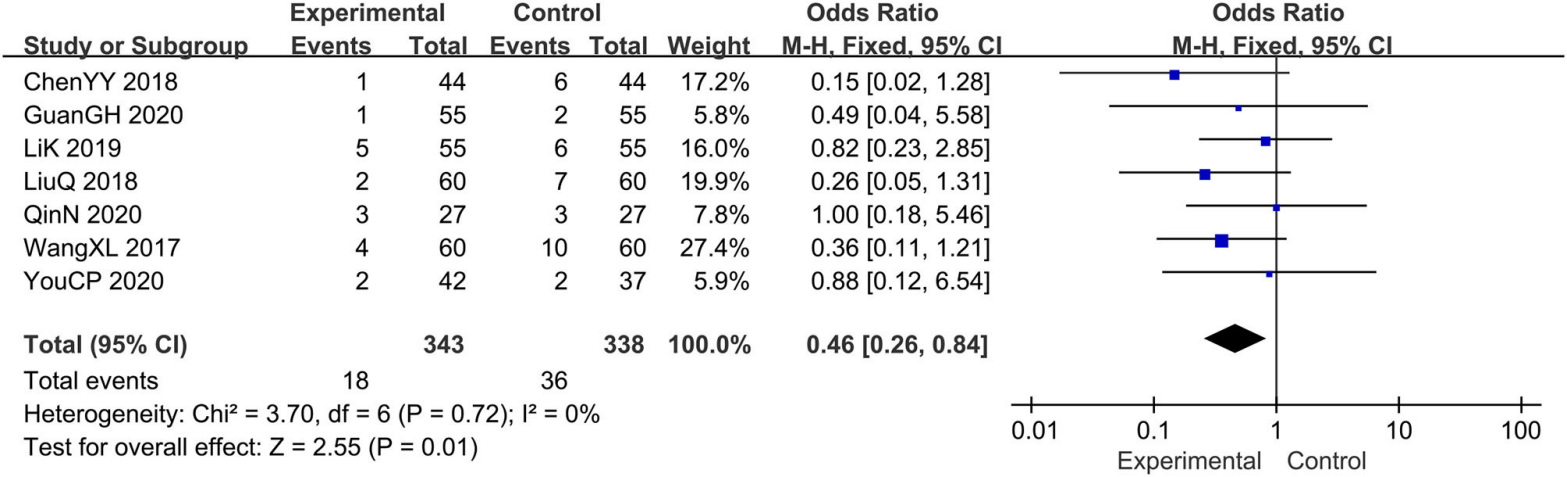
The meta-analysis for comparison of combination therapy vs. prednisone for the adverse events.

Seven trials evaluated the adverse events (14, 16, 18–21, 23), comprising a total of 681 patients. The fixed-effect model was adopted due to the low heterogeneity (*I*^2^ = 0%, *p*=0.72). Results showed that relative to the prednisone group, combined with tanshinone significantly reduced the adverse events, OR: 0.46; 95% CI: 0.26, 0.84; *p* = 0.01 (Figure 9).

### The Recurrence Rate

**Figure 10:**
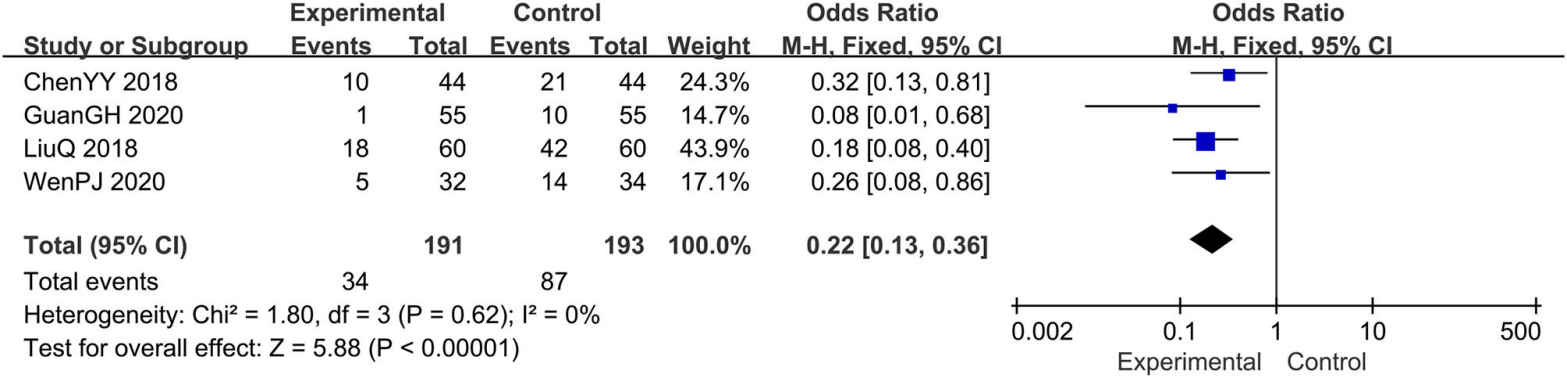
The meta-analysis for comparison of combination therapy vs. prednisone for the recurrence rate.

Four trials evaluated the recurrence rate (14, 15, 18, 19), comprising a total of 384 patients. The fixed-effect model was adopted due to the low heterogeneity (*I*^2^ = 0%, *p* = 0.62). Results showed that, relative to the prednisone group, the combination therapy group significantly reduced the recurrence rate, OR: 0.22; 95% CI: 0.13, 0.36; *p* < 0.00001 (Figure 10).

### Evaluation of Publication Bias

**Figure 11:**
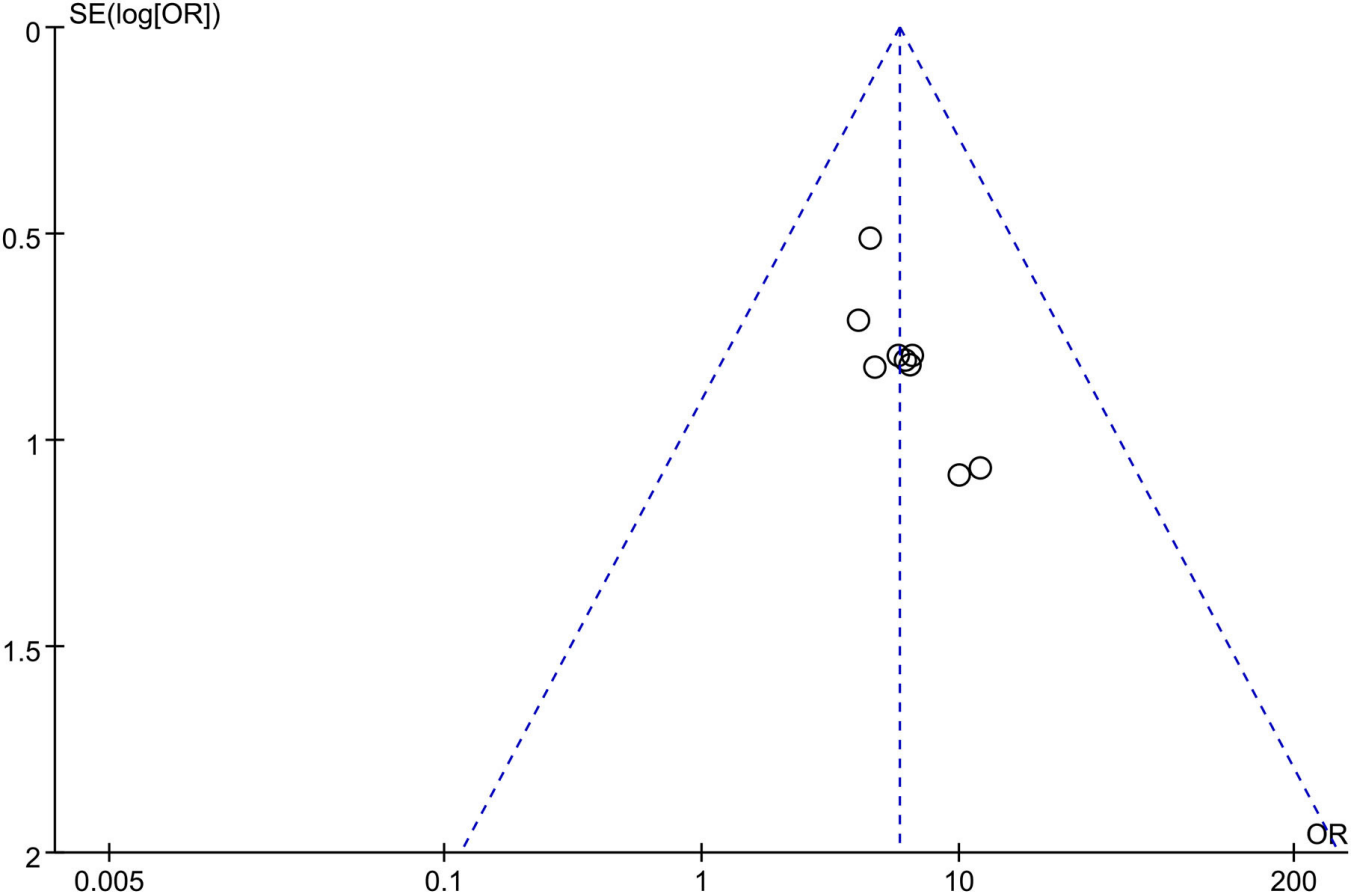
Funnel plot of the clinical efficacy rates: combination therapy vs. prednisone.

Based on the clinical efficacy rates, we used Review Manager Software 5.3 to evaluate publication bias. The funnel plot showed that all research projects are roughly concentrated and symmetrical, indicating that the remaining studies may have less publication bias (Figure 11).

## Discussion

Although the pathophysiological conditions of FSD are currently unknown, most sources agree that Malassezia colonization, sebaceous gland hypersecretion, and underlying immune system susceptibility are the main issues affecting patients (24). Malassezia plays a dominant role in the microbiome of seborrheic dermatitis and is the main cause of the disease (25), the density of Malassezia was positively correlated with inflammation (26). Malassezia is lipid-dependent yeasts that can hydrolyze free fatty acids, activate the immune system through pattern recognition receptors, inflammatory corpuscles, and NF-KB pathways, which produce an inflammatory response, destroy the skin barrier, by forming a biofilm to block the action of the drug, and further develop drug resistance (27). The hypersecretion of sebaceous glands can directly provide nutrition for Malassezia, while the parasite of Malassezia can change the proportion of lipid components in the scalp and damage the skin barrier (28). However, not all patients with a high density of Malassezia develop the disease, so it may be possible that dysregulation of the immune system is required for their detrimental action (29).In addition, abnormal trace elements (30), epidermal microbial abnormalities (31, 32), and genetic predisposition (33) also affect the occurrence of FSD.

Topical therapies are the basic therapy for FSD, antifungal agents, anti-inflammatories, and keratolytic are considered to be the first-line of therapy for the management of FSD (34). Triamcinolone Acetonide and Econazole Nitrate Cream (*Trade name: Pevisone*) contains triamcinolone acetonide and econazole, Triamcinolone acetonide is a medium and long-acting glucocorticoid with anti-inflammatory and antipruritic actions, econazole is an azole antifungal that interferes with the biosynthesis of fungal cell membranes and inhibits ribonucleic acid synthesis, meanwhile, they have activity against some gram-positive bacteria in addition to their broad-spectrum antifungal. Steroids and azoles were equally effective in producing total clearance of lesions of FSD (35). The most common adverse effects were redness, burning, and itching at the application site (36). Tanshinone is a natural terpenoid, which is the mainly fat-soluble bioactive component extract from *Salvia miltiorrhiza Bunge [Lamiaceae]*, it contains tanshinone I, tanshinone IIA, dihydrotanshinone, and cryptotanshinone (37, 38). Tanshinone capsules can treat FSD through the following mechanism. Firstly, tanshinone has an anti-androgen effect and estrogen activity, which can regulate skin endocrine and metabolic function, and maintain hormone balance (39, 40). Secondly, tanshinone capsules have a broad-spectrum antibacterial effects, such as hydroxytanshinone and methyl tanshinate can kill and inhibit a variety of aerobic and anaerobic bacteria, especially can inhibit gram-positive bacteria (41, 42). Furthermore, it also has anti-inflammatory effects by inhibiting the release of oxygen free radicals by white blood cells and reducing the levels of PGF2a and PGE in the blood. In addition, the tanshinone capsules can also inhibit the proliferation of sebaceous glands by blocking androgen receptor expression in sebaceous gland cells to resist androgen action and reduce lipid synthesis and secretion (43). Beyond that, the tanshinone capsule also has an anticoagulant effect, improves facial blood circulation, and promotes skin metabolism. Ultimately, it has extensive immunomodulatory effects and plays a significant role in the development and function of immune cells (44). The oral tanshinone capsule can be quickly absorbed by the gastrointestinal tract and distributed throughout the body. It has the characteristics of fast-acting and slow excretion. Combined with triamcinolone and Econazole cream, it can coordinate internal and external recuperation, jointly promote the regression of skin lesions and make the skin return to a normal state.

Therefore, this study systematically evaluates the safety and effectiveness of tanshinone capsules combined with Pevisone in the treatment of FSD by collecting RCTs. The results show that combination therapy can improve clinical efficiency, and reduce the scores of symptoms such as erythema, itchy, furfuraceous desquamation, and lesions. Moreover, combination therapy can reduce adverse events and control recurrence rates. However, some limitations of this study should be acknowledged. Firstly, we did not discuss hematological or immunological indicators because the included clinical randomized controlled trials did not provide sufficient laboratory testing indicators. Secondly, we were unable to obtain the specific design of some trials, which makes it impossible to evaluate the literature quality, and the quality of individual trials may affect the reliability of the study.

## Conclusion

In conclusion, tanshinone capsules combined with Pevisone are effective in the treatment of FSD. Further large-scale RCTs and more objective observational indicators should be conducted to assess the tanshinone capsules in FSD.

## Data Availability Statement

The original contributions presented in the study are included in the article/Supplementary Material, further inquiries can be directed to the corresponding author/s.

## Author Contributions

QF and MH conceptualized and designed the study and reviewed the protocol for important intellectual content. QF, LT, and QZ drafted the manuscript and collected data. QF and FH analyzed the data. It has been edited by SW and XZ as native. All authors read and approved the final manuscript.

## Funding

This study was supported by the Chongqing City Science and Technology Plan Project (No. 2021ZY023609), the National Science Fund for Distinguished Young Scholars of China (Grant No. 82104872), the Xinglin Project of School of Traditional Chinese Medicine, and Chongqing Medical University (No. 2021-ZDXK-D02).

## Conflict of Interest

The authors declare that the research was conducted in the absence of any commercial or financial relationships that could be construed as a potential conflict of interest.

## Publisher's Note

All claims expressed in this article are solely those of the authors and do not necessarily represent those of their affiliated organizations, or those of the publisher, the editors and the reviewers. Any product that may be evaluated in this article, or claim that may be made by its manufacturer, is not guaranteed or endorsed by the publisher.
