# Supplementary material for: Tanshinone Capsules Combined With Prednisone for Facial Seborrheic Dermatitis: A Systematic Review and Meta-Analysis of Randomized Clinical Trials
Source: Front Med (Lausanne). 2022 Apr 29;9:816419. doi: 10.3389/fmed.2022.816419 (PMC9106380; doi:10.3389/fmed.2022.816419)
Supplement: Supplementary file 1 [file Table_1.docx]

| **SUMMARY TABLE OF THE STUDIES INCLUDED**. | | | | | |
| --- | --- | --- | --- | --- | --- |
| **Study** | **Formulation** | **Source** | **Species, concentration** | **Quality control reported**  **(Y/N)** | **Chemical analysis reported**  **(Y/N)** |
| GuanGH et al.(2020) | Tanshinone capsules | Hebei xinglong Xili Pharmaceutical Co., Ltd | Ethanol extract from Root of *Salvia miltiorrhiza Bunge*. [*Labiatae; Salviae Miltiorrhizae*], 0.25g | Y – Prepared according to the Pharmacopoeia of China, 2020 edition | N |
| WenPJ et al.(2020) | Tanshinone capsules | Hebei xinglong Xili Pharmaceutical Co., Ltd | Ethanol extract from Root of *Salvia miltiorrhiza Bunge*. [*Labiatae; Salviae Miltiorrhizae*], 0.25g | Y – Prepared according to the Pharmacopoeia of China, 2020 edition | N |
| LiK et al.(2019) | Tanshinone capsules | Hebei xinglong Xili Pharmaceutical Co., Ltd | Ethanol extract from Root of *Salvia miltiorrhiza Bunge*. [*Labiatae; Salviae Miltiorrhizae*], 0.25g | Y – Prepared according to the Pharmacopoeia of China, 2020 edition | N |
| WangF et al.(2019) | Tanshinone capsules | Hebei xinglong Xili Pharmaceutical Co., Ltd | Ethanol extract from Root of *Salvia miltiorrhiza Bunge*. [*Labiatae; Salviae Miltiorrhizae*], 0.25g | Y – Prepared according to the Pharmacopoeia of China, 2020 edition | N |
| ChenYY et al.(2018) | Tanshinone capsules | Hebei xinglong Xili Pharmaceutical Co., Ltd | Ethanol extract from Root of *Salvia miltiorrhiza Bunge*. [*Labiatae; Salviae Miltiorrhizae*], 0.25g | Y – Prepared according to the Pharmacopoeia of China, 2020 edition | N |
| LiuQ et al.(2018) | Tanshinone capsules | Hebei xinglong Xili Pharmaceutical Co., Ltd | Ethanol extract from Root of *Salvia miltiorrhiza Bunge*. [*Labiatae; Salviae Miltiorrhizae*], 0.25g | Y – Prepared according to the Pharmacopoeia of China, 2020 edition | N |
| QinN et al.(2020) | Tanshinone capsules | Hebei xinglong Xili Pharmaceutical Co., Ltd | Ethanol extract from Root of *Salvia miltiorrhiza Bunge*. [*Labiatae; Salviae Miltiorrhizae*], 0.25g | Y – Prepared according to the Pharmacopoeia of China, 2020 edition | N |
| WangXL et al.(2017) | Tanshinone capsules | Hebei xinglong Xili Pharmaceutical Co., Ltd | Ethanol extract from Root of *Salvia miltiorrhiza Bunge*. [*Labiatae; Salviae Miltiorrhizae*], 0.25g | Y – Prepared according to the Pharmacopoeia of China, 2020 edition | N |
| LuDD et al.(2021) | Tanshinone capsules | Hebei xinglong Xili Pharmaceutical Co., Ltd | Ethanol extract from Root of *Salvia miltiorrhiza Bunge*. [*Labiatae; Salviae Miltiorrhizae*], 0.25g | Y – Prepared according to the Pharmacopoeia of China, 2020 edition | N |
| YouCP et al.(2020) | Tanshinone capsules | Hebei xinglong Xili Pharmaceutical Co., Ltd | Ethanol extract from Root of *Salvia miltiorrhiza Bunge*. [*Labiatae; Salviae Miltiorrhizae*], 0.25g | Y – Prepared according to the Pharmacopoeia of China, 2020 edition | N |
